# Effects of vaccine registration on disease prophylaxis: a systematic review

**DOI:** 10.1186/s12938-022-01053-z

**Published:** 2022-12-03

**Authors:** Suelia de Siqueira Rodrigues Fleury Rosa, Ana Karoline Almeida da Silva, Carolina Ramos dos Santos, Mayla dos Santos Silva, Ana Luísa Pereira Perillo, Arthur Faria Mendonça, Mario Fabrício Fleury Rosa, Thatiane Lima Sampaio, Marcella Lemos Brettas Carneiro, José Carlos Tatmatsu Rocha, Antônio Piratelli-Filho, Allisson Lopes de Oliveira

**Affiliations:** 1 Biomedical Engineering Program at Faculty of Gama, University of Brasília, Brasília, DF Brazil; 2 Center for Research and Technological Innovations in Human Rehabilitation – INOVAFISIO, University Federal of Ceará, Fortaleza, Ceará Brazil; 3 Mechatronic Systems Program, at Mechanical Engineering Department, University of Brasília, Brasília, DF Brazil; 4 University Center of Mineiros, Goiânia, GO Brazil; 5 University of Rio Verde, Goiânia, GO Brazil; 6 Federal Institute of Education, Science and Technology, Brasília, DF Brazil; 7 Graduate Program in Medical Sciences, Faculty of Medicine, University of Brasília, Brasília, DF Brazil

**Keywords:** Vaccination, Mobile applications, Health smarts cards, Immunization programs, Vaccination coverage

## Abstract

**Background:**

The impact of the pandemic caused by the coronavirus (SARS-CoV-2), causing the disease COVID-19, has brought losses to the world in terms of deaths, economic and health problems. The expected return of the public to activities adapted to the new health situation led to discussions about the use of vaccination and its effects. However, the demand for proof of vaccination showed how inconsistent, unregistered, and uncontrolled this health process is with current technologies. Despite the proven effectiveness of vaccines in reducing infection rates, mortality, and morbidity, there are still doubts about their use in preventing certain infections and injuries, as well as the use of digital medical records for identification at public events and disease prevention. Therefore, this review aims to analyze the use of digital immunization cards in disease prevention in general.

**Methods:**

A systematic review of Science, PubMed/MEDLINE, LILACS /BSV, CINALH, and IEEE and Xplore was performed using PRISMA guidelines. The authors summarized the studies conducted over the last decade on the impacts of prophylaxis by control through immunization cards. Studies were selected using the following terms: Vaccination; Mobile Applications; Health Smarts Cards; Immunization Programs; Vaccination Coverage. For data analysis, we used Mendeley, Excel, RStudio, and Bibliometrix software among others.

**Results:**

A total of 1828 publications were found. After applying eligibility criteria (Articles published in Portuguese, Spanish or English in the last 10 years). Studies that only dealt with paper or physical records were excluded, as well as studies that were not linked to their country’s health Department, as a possibility of bias exists with these types of information). After removing duplicates and applying filters 1 and 2, we included 18 studies in this review. This resulted in 18 papers that met our priori inclusion criteria; it was found that the most relevant sources were from the databases of the Institute of Electrical and Electronics Engineers (IEEE).

**Conclusions:**

Considering the selected studies, we found that scientific evidence and epidemiological surveillance are essential tools to characterize the efficiency and effectiveness of immunization passport protection intervention and to ethically justify them. Technological development of digital vaccine passports can assist in vaccination programs and positively impact disease prophylaxis.

**Supplementary Information:**

The online version contains supplementary material available at 10.1186/s12938-022-01053-z.

## Background

The pandemic caused by the SARS-CoV-2 coronavirus, which causes COVID-19, has led health authorities worldwide to propose various forms of social behavior modification based on idea of limiting contagion, based on the understanding that some pathogenic biological entities can spread rapidly between individuals. One of the most important actions has been vaccination, which has always been a principal public health tool for preventing and controlling diseases, including the SARS-CoV-2 virus, due to its case of airborne transmission [1].

The virus’s rapid spread in some countries was contributed to the influx of people from China to other countries. This factor prompted the World Health Organization (WHO) to declare COVID-19 as global health emergency. The growing number of cases in many countries led to the disease officially being declared a pandemic. The disease has spread to more than 160 countries, and there were more than 492,357,252 people infected at the time of this writing. The speed of the spread of the disease culminated in a warning from the World Health Organization (WHO), which named the emerging disease COVID-19 and labeled it a pandemic and a health problem of international risk and emergency character [2]. Therefore, one of the biggest challenges for government agencies is to slow the spread of the virus and prevent the collapse of the health care systems [3]. The disease spread out to more than 160 countries and infected more than 1 million people worldwide. Estimates of severe cases are the main concern of health authorities worldwide [1, 2].

The primary clinical manifestations of COVID-19 are fever (90% or more), cough (about 75%) and dyspnea (up to 50%) [2]. These findings are corroborated by other studies, including the study by Yang et al. [1], which also reported changes in computed tomography images of the lungs that indicate more opacification, particularly in the lower lobes of the lungs. However, they emphasize that such findings may be heterogeneous, varying from person to person or even absent [3, 4]. Mild symptoms include headache or dizziness, diarrhoea, nausea and vomiting [4].

Traditionally, the vaccination card has been printed on paper cards that records the vaccinations that patients received at health care facilities. The information on these cards generally provides details about the person who received the dose, the date of administration, the type of vaccine, and other essential information added by professionals [5]. Digital vaccination cards serve the same purpose of ensuring continuity of care and providing proof of vaccination. The professionals responsible for the recording and the patient have access to this documentation. Some countries require an International Certificate of Vaccination or Prophylaxis (ICVP), which certifies vaccination against diseases that may occur as a result of international travel, e.g., the yellow fever vaccine [6].

Presently, it is possible for a vaccination card to be completely digital, eliminating the need for a physical (paper) identity (ID) card. The World Health Organization recommends that digital certificates do not require electronic resources from the population, such as smartphones or computers [7].

The digital immunization (vaccination) card allows users and health professionals to track immunizations. The user can download the app, register, and once they receive the vaccine, the dose is automatically registered on the app by the healthcare professional. The card allows the user to retrieve the type of vaccine used, the manufacturing batch, and the date it was taken. These technologies have already been used at sporting events in Europe and Brazil [8].

The risk scenario to populations created by the new coronavirus pandemic required Unified Health System (SUS) of Brazil, to coordinate national efforts and orchestrate the actions of states, communities, and additional health services. Therefore, after the National Health Data Network (NHDN) launch, Conecte SUS Citizen app was refocused on obtaining and sharing information to help control Brazilian public health emergencies, focusing on the COVID-19 vaccination process [6]. Brazilian citizens can view interactions carried out in health centers and follow their progress on SUS, such as examinations, vaccinations, drug dispensing, and health facility locations [5, 7].

In response to the COVID-19 pandemic, there has been much discussion about vaccination passports. Some have argued that immunity passports are unethical and impractical. They point to uncertainties surrounding COVID-19 immunity, problems with testing, uncertain economic benefits, privacy concerns, and risk of discriminatory effects [9].

Immunity passports can be introduced based on laboratory immune response testing (a correlation of protection) or an immunization event (infection or vaccination) and identify individuals who are less likely to be either infected with SARS-CoV-2 or to transmit the virus. Scientific evidence and epidemiologic surveillance are essential tools for characterizing and thus ethically justifying the efficiency and effectiveness of protective measures based on a vaccination record. For example, in July 2021, the European Union Commission launched a digital vaccination passport program to revitalize economic, social, and cultural activities [10].

Some countries require a Certificate of International Vaccination or Prophylaxis (CIVP) for international travel that proves vaccination protection against disease. This requirement can be found under the Travel Requirements tab. The yellow fever CIVP is required by more than 100 countries [11]. The CIVPs for yellow fever, polio, meningitis, and COVID-19 are issued in Brazil by the National Health Administration (ANVISA) [6]. A vaccination card can be purely digital (e.g., stored on a smartphone app or cloud-based server) and replace the paper card, or it can be a digital representation of the traditional paper document.

The WHO has standardized nomenclature for digital documentation of COVID-19 certification: vaccination Status (DDCC:ES), from the English “Digital Documentation of COVID-19 Certificates: Vaccination Status (DDCC:VS)” [12]. The WHO recommends that a digital certificate should never require an individual to own a smartphone or computer. For example, the link between the paper record and the digital record can be established via a one-dimensional (1D) or two-dimensional (2D) barcode printed on or attached to the paper vaccination certificate [12, 13]. When this document refers to “paper”, it means a physical document (printed on paper, plastic card, cardboard, etc.).

The measures used to contain the virus are mass vaccination of the population, and the successful adoption of vaccination strategies has already been observed in several countries. However, analyzing the impact of prophylaxis by reviewing vaccination results is a strategy that must be scientifically sound. Health technology tools, in the form of applications can help us answer the question of whether digital verification of vaccination results is an effective strategy for disease prophylaxis and improving vaccination coverage and disease control [5]. The purpose of this study is to review the published literature to assess the impact of digital management of immunization records on global disease prevention. In addition, this study seeks to review the published literature to assess the impact of vaccination records on prevention of some diseases. This article is a systematic review that summarizes findings relevant to translational studies. The meanings of the acronyms can be found in Additional file 5: Glossary

## Results

**Fig. 1:**
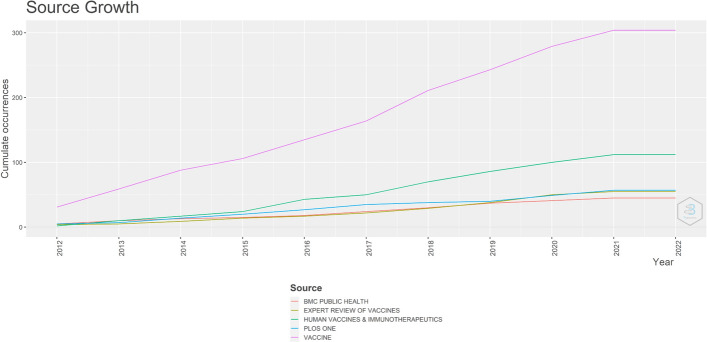
Main journals that published in the area of this systematic review. They were developed using RStudio, using the Bibliometrix package

A bibliometric analysis of 02/20/2022 in the databases using the search terms (“Vaccination” AND “Mobile Applications” OR “Health Smarts Cards” OR “Immunization Programs” OR “Vaccination Coverage” OR “International Certificate of Vaccination” AND “Prophylaxis”) found 1828 publications, with duplicates excluded. The search was performed using RStudio software, and the data were analyzed using Bibliometrix [14]. In Fig. 1, we show the distribution of production across years. In 2019/2020, especially during the pandemic COVID-19, there was an increased search for technological solutions that would allow for improved disease prophylaxis.

### Characteristics of included studies

**Fig. 2:**
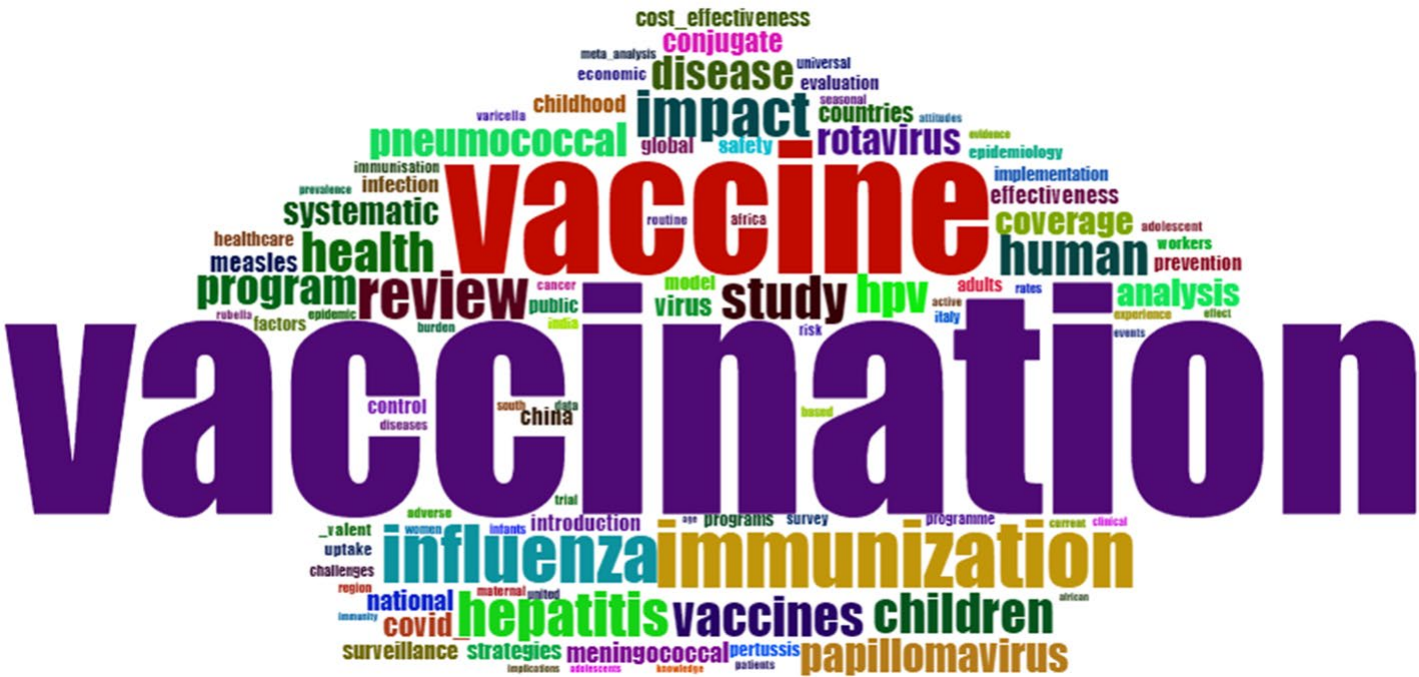
Word Cloud. Bibliometric analysis, with the RStudio through the Biliometrix, of the 1828 works published with the search string (“Vaccination” AND “Mobile Applications” OR “Health Smarts Cards” OR “Immunization Programs” OR “Vaccination Coverage” OR “International Certificate of Vaccination” AND “Prophylaxis”), in title/abstract/words-keys, on xxx, march 2022, with a minimum co-occurrence of terms of 20 times [14]

Using a bibliometric analysis of the terms in this study, we can analyze the frequency of occurrence of terms in publications, Fig. 2. The word cloud includes 100 terms, with the most prominent, “vaccination,” occurring 759 times.

**Fig. 3:**
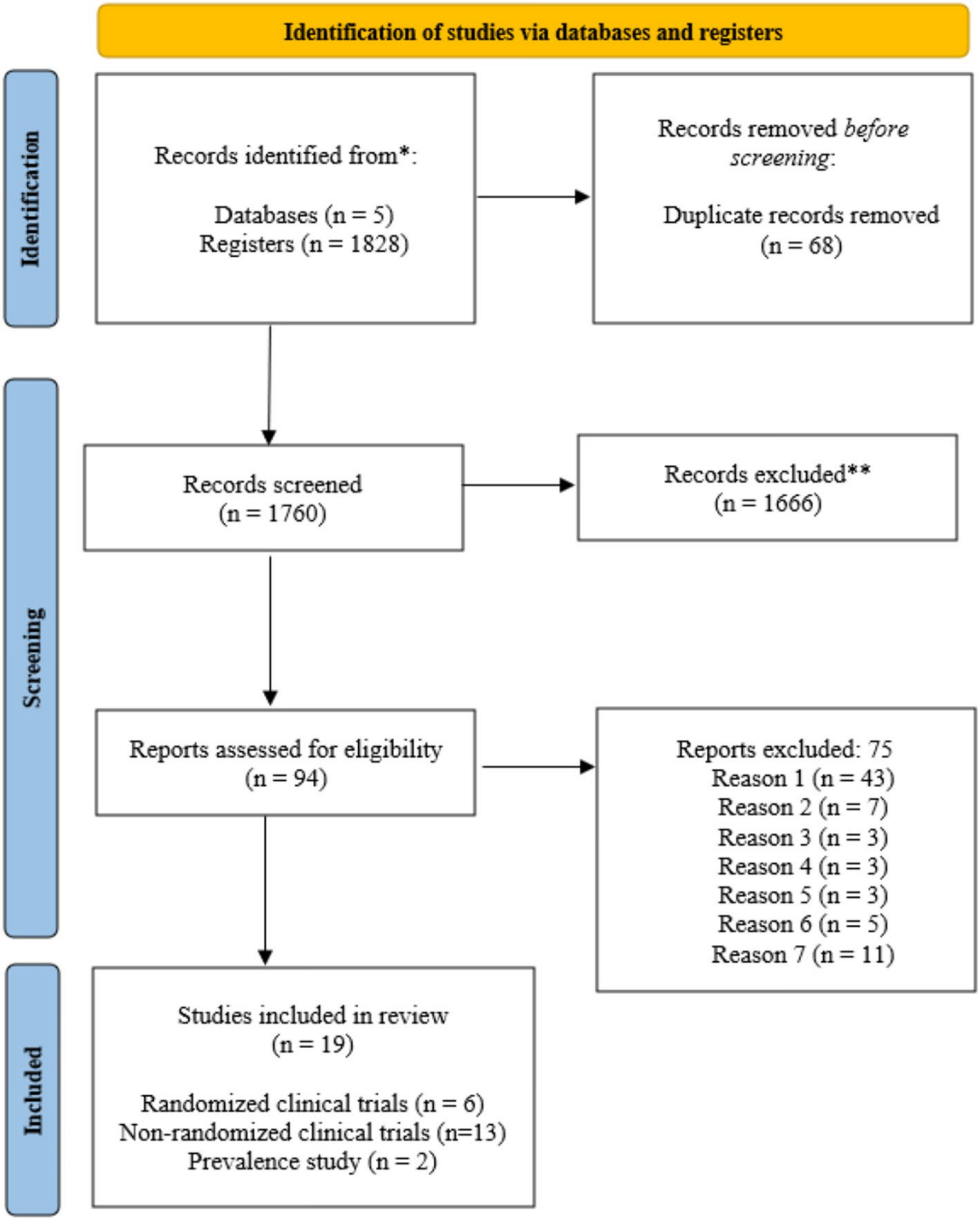
PRISMA 2020 flow diagram for new systematic reviews which included searches of databases and registers only. From: Page et al. [15]. For more information, visit: http://www.prisma-statement.org/

After using the comprehensive search string, we identified 1828 citations by searching the selected databases and listing relevant article references. After removing duplicates, 1760 entries were found. Articles were selected according to the PRISMA flowchart, Fig. 3. This resulted in 18 papers that met our a priori inclusion criteria.

**Fig. 4:**
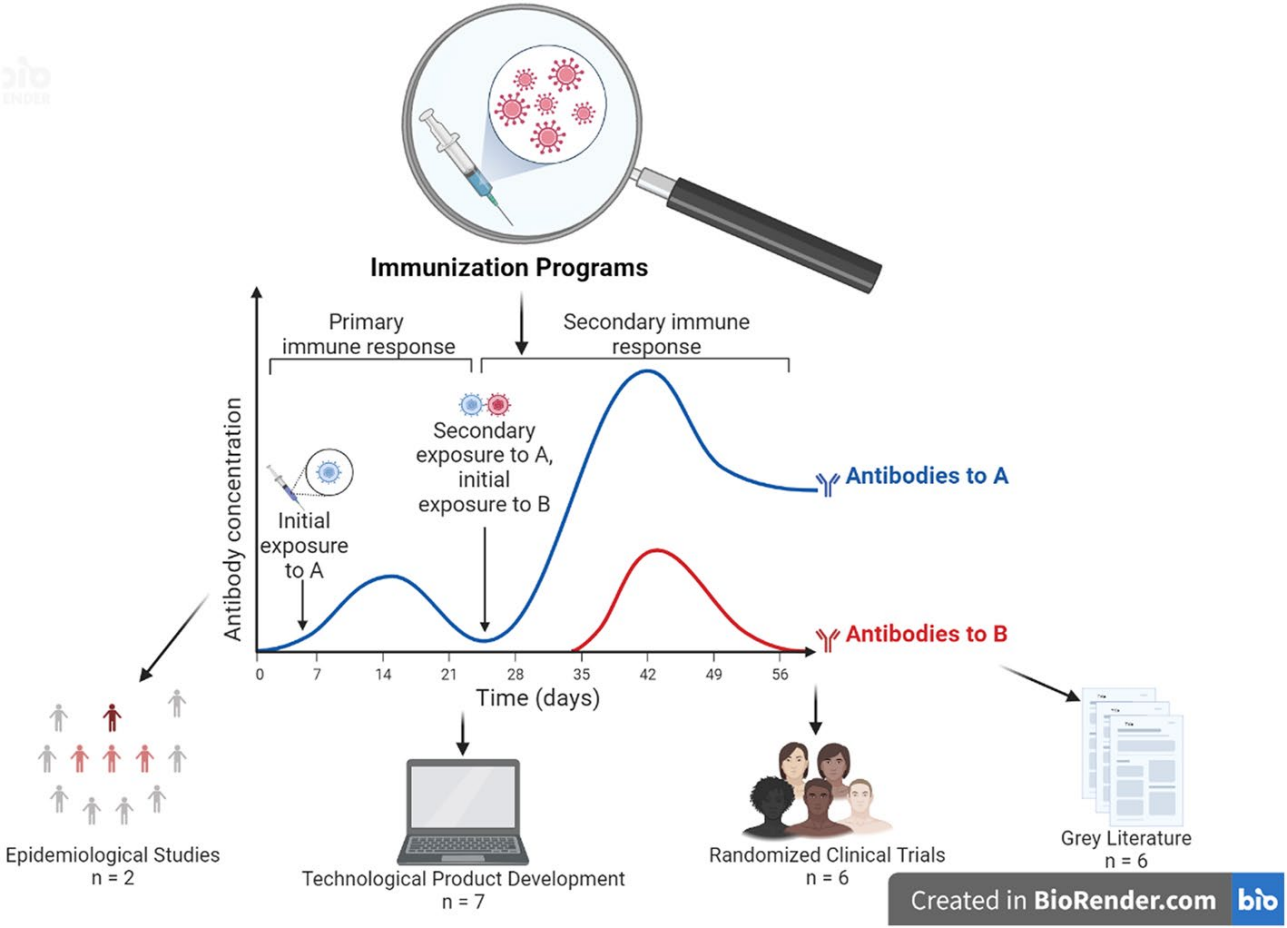
Categories of studies selected for this review. Created in Biorender software (2022) [16]

As this assessment relates to technological development, it was necessary to divide the references into 4 categories for data extraction and risk of bias analysis, as shown in Fig. 4, namely—(I) technological products (*n* = 7); (II) epidemiological studies (*n* = 2), (III) randomized clinical trials (*n* = 6), and (IV) gray literature (*n* = 4). The characteristics of the included studies are listed in Additional file 4.

Of the articles selected for this review, seven studies were conducted in the United States [17–23], one in Italy [24], two in Iran [25, 26], and one in Brazil [27], Canada [28], India [12], South Korea [13], Australia [29], Africa [30], Malaysia [31], Nigeria [32], and Bangladesh [33]. Three studies addressed the HPV vaccine [22, 23, 29], influenza vaccine [19–21], and oral cholera vaccine [33]. Approximately 11 studies addressed data extraction, manipulation, interpretation, and analysis [13, 17, 21–23, 25, 26, 28–30, 32]. The study population included the general population, a fictitious population, and sensitive data.

Of the studies associated with randomized clinical trials, 50% were conducted from 2019 to 2021. Research addressed strategies for immunization against influenza (*n* = 3) [19–21], HPV (*n* = 2) [22, 23], Tdap, and pneumococcus (*n* = 1) [20]. The heterogeneity of the studies allowed for analysis of the results with samples in different groups of adults (*n* = 2) [20, 21], adolescents (*n* = 3) [19, 22, 23], and children (*n* = 1) [32].

The six randomized controlled trials used vaccination reminder apps and systems via text messages and e-mails. Immunization monitoring and electronic communication were used to increase rates of adherence to immunization programs, which was statistically significant in the studies of [19, 21, 32]. Messages to voicemail or automatic dialing did not achieve significant results [22, 23], although they were associated with lower implementation costs at an average cost per patient of $0.86—compared with real-time calls—an average dollar cost of 0.5 cents per call [32]. The authors [29] also used a text messaging service with shared motivational messages and reminders. Both increased vaccination rates among 7 years, but there were no statistical differences between them.

The use of telephone calls was an option, and studies [25, 26] evaluated the effectiveness and cost of implementing automatically dialed telephone messages. In Ershadi et al. [26], and immunization registry platform was established for the first time in the Iranian health system, focusing on medical personnel. It was shown that the analytical capacity of the system can be an approach to promote better management policies and procedures in controlling immunization at the national level. In Jadidi et al. [25], electronic vaccination records were found to increase vaccination coverage. Through the reminder function, this has an impact on public health and outbreak control. The important implications of using technology to support reminder-based practices continues to evolve rapidly, and resources to effectively track and promote achievement of such goals can be validated with clinical trials that relate targeted reminders and program goals.

Messages left in the mailbox are considered part of an old system and are sometimes less utilized by users. In the study conducted by [23], the authors mention the difficulty of obtaining valid numbers, which reduced the sample size, while [20] showed an acceptance of 91% (16,408/17,951) of users to receive automatic dialing messages. In a study of children, 99.2% of responsible parties agreed to receive reminder messages, with the mother representing the vast majority (98% of the sample) [32]. The use of telephone calls was an option, and studies [19, 26] evaluated the effectiveness and cost of implementing autodialed telephone messages. In Ajakwe et al. [13], electronic immunization records were found to increase vaccination coverage. This has implications for public health and disease outbreak control. The important implications of using technology to support reminder-based practices continue to evolve rapidly, and resources to effectively track and promote achievement of such goals can be validated with clinical trials that correlate targeted reminder and program goals.

E-mail communication systems were also analyzed. The authors used a reminder system sent by e-mail after vaccination status was verified through local health system software and reminder letters sent each influenza season. The results obtained were not significant in both season 1 (*p* = 0.06) and season 2 (39% vs. 37%, *p* = 0.20) [19]. The status analyses on immunization status were the Michigan Care Improvement Registry (MCR) [19], CIIS [20, 23], Human Welfare [21], MCO [22]. Of these, only 1 focused on the private health insurance system [21], and 1 used paper records [32]. Through these applications, researchers obtained contact information and the immunization schedule, and later contacted them to obtain consent to participate in the study. However, the lack of updating of registered contacts was a limitation for the authors [19, 20, 22, 23]. Of the studies that addressed the development of technological solutions to monitor and promote a comprehensive vaccination program, 57.14% were from 2020/2021, indicating that COVID-19 was driving this search for technological solutions to expand vaccination. For studies linked to epidemiological studies, we have a total of 60% of studies conducted in pandemic years (2019/2020) with the search for tools to help demonstrate the impact of partnerships with vaccine providers to evaluate the response to the pandemic through vaccination. Geographic area identification and mapping strategies were used to generate data on vaccination activities in segments of field areas that facilitate vaccine delivery and to identify socioeconomic determinants of vaccine uptake in urban areas.

Among the studies linked to the gray literature, 75% of the studies presented were from 2019 and only one was from 2020. These studies considered vaccination as a form of public health intervention in the context of disease prevention and specifically emphasized vaccination resistance, which is related to multiple and complex factors. Subsequent studies [26, 30, 31] have used the Web platform, developed and adapted an electronic immunization data system, and demonstrated that collection and electronic storage provide more accurate data, greater precision and reliability, faster access, and greater security. The impact on immunizations was evaluated from the point of view of the comprehensive functions of the software implementing the procedure, the data to be compared, and the evaluation of immunization protection. Another observed impact is the lack of control of immunization records—the use of the one-page paper structure, which affects the real flow of information, as it is not possible to record in detail the impact of diseases and link them to the absence of immunizations.

Despite the increasing use of applications in healthcare, only 16.67% of productions in [12, 13, 18] use application tools and only three reviewed studies reported the use of technologies through QR-code reading and wireless sensor systems [13, 17, 31]. In Hasan et al. [12], a literature search was conducted by searching some digital libraries such as IEEE, Springer and J-Gate with some specific keywords such as vaccine, mobile app, vaccine management, etc. Over the years, a total of 20 articles on vaccine management websites and apps were selected. They served as the basis for the development of the app to facilitate the vaccination process and help parents and caregivers better adhere to their children’s vaccination schedules. In Katib et al. [18], the target audience was a rural setting, a software application called Jeev Para with two important functions, one for tracking and recording information about immunizations. Although the application and testing were limited to environments such as a large rural setting, this solution can provide coverage for children ensuring consistent immunization. Regarding the application’s country of focus, it should be noted that in Haiti, as in other countries, cell phones and short message system (SMS) messaging have become very popular. The Haitians mainly use for mobile banking. Other data that can be measured are school dropout rates of these families. In Ajakwe et al. [13], a convergent approach was developed for integration of information technology (IT) and a compliance system for real-time. The monitoring and enforcement of COVID-19 through immunization and prevention policies using facial recognition, facial mask recognition technology, a system to manage immunization information and status of vaccine validation requests using QR code authentication technology. It is noteworthy that the system has achieved 99.5% real-time recognition, classification, validation, authentication, and application accuracy with less computational complexity and with implementation and integration. It is believed to have reduced the spread and transmission of viruses, among other intangible benefits, but there is no clinical study to validate these data.

In Sun et al. [17], a wireless sensor system was developed to map student contacts related to the spread of disease over distances and to generate graphs of disease spread that can assess transmission of infectious diseases in an environment with 800 students. The proposal also highlights that the connectivity–centrality metric measures the importance of a node during disease spread and develops centrality-based algorithms for targeted vaccination. In this study, the infection rate changes over time with $\alpha$ = 2.5% and 10%, respectively. The spread of the disease was divided into three phases. First, the disease spreads slowly from the infection sources, then it spreads widely and the infection rate increases rapidly, and finally the infection rate remains stable.

In Fiquaro et al. [31], the creation of a blockchain immunization system contributed, significantly with functionalities that can be added in the future—however, the registration and collection of important information are already being done. Two important tests were conducted, a so-called black-box test to test system dynamics (input and output), and another white-box test to analyze the flow of the solution construction. The results of the first test—with a simple user accessing the network and logging in with an administrator address—showed a good response from the website in terms of loading, routing and the hospital system interface. In the black-box test, vaccination data entry and correlation with the algorithm worked flawlessly. For example, when the patient identification was missing (not registered), the algorithm redirected to the patient registration form page.

In Paula [27] in the dissertation conducted in Brazil, they showed that a total of 27 evaluated apps, the size on Android platform ranged from 0.95 to 81.06 (21.6) and on iOS from 1.02 to 35.92 (20.3), developed between the release in 2012 and 2018. We were unable to capture the highlights we observed that most apps (*n* = 19) were only available in English and were updated in 2017 or 2018 (*n* = 20) as these apps were published and validated in randomized clinical trials. An app review result showed that the average user rating for the Android platforms was 3.9 out of 5.0. No user ratings were found for the iOS platform, because the virtual AppStore does not offer this feature. One point that stood out was that the apps with the highest ratings were: CANimmunize and Vaccine Reminer, Babá Digital, and The Vaccine Handbook app. None of these apps perform prophylaxis monitoring or provide data feedback for that monitoring. The app called Proimuni has developed an application for newborns that highlights the importance of recording vaccinations since time of birth.

In Moonsamy [30], the authors found no fully automated system for managing immunization records with implications for prophylaxis in a review of the literature. The reported results of the survey showed that there are benefits to using a digital system to store and manage immunization records. Another highlight is that of the 5 BRICS countries. Of the 5 only 2, Brazil and Russia are still hesitant to use mixed systems. The study shows a great need to build and clinically test an integrated national telemedicine system for immunization to capture the impact of prophylaxis, hospitalization, and other parameters.

The results demonstrate the high complexity and dynamics of telemedicine application development involving software engineering. The creation and improvement of public policies and the flow of information as transparent as possible are necessary. It is critical to contributing to public health security, public safety, better use of public resources, and greater consistency in the effectiveness of the response to pandemics, endemic diseases or outbreaks.

In the epidemiological studies, they developed mechanisms for vaccination campaign planning and real-time monitoring of individuals’ vaccination coverage using the software ERDAS IMAGINE, which utilizes global positioning data (GPS) for georeferencing [33]. They subdivided the Mipur–Bangladesh region into clusters and considered socioeconomic factors in data analysis. They used vaccines from different manufacturers and mass vaccination campaigns. Considering socioeconomic factors and mapping vaccination areas may help eradicate the disease. The impact of vaccination on children in each location and the corresponding mortality and morbidity rates were analyzed by Wilson et al. [28]. For ethical reasons, the study did not report specific sample data, statistical results, or percentages of adverse events. However, it showed that the use of similar software could analyze the effects of mass vaccination. Below are Tables 1, 2, 3 and 4 with the excerpts.

**Table 1:** Data extraction from epidemiological studies

Study	Population	Intervention			Outcomes	
Author, year/country	Sample	Pathology	Instruments	Methodology	Prophylaxis	Technological progress in the health system
Saha et al., 2018, Bangladesh	Inhabitants of the city of Mipur (*n* =268,896), divided by clusters and 3 study arms. G1 vaccination (*n* = 95,115); G2 vaccination and behavior change (*n* = 93.09); G3 No intervention (*n* = 80,690).	Cholera	ERDAS IMAGINE software-geographic-monitoring-system	Once the areas to be vaccinated were mapped using ERDAS, the socioeconomic profile of the population was determined. Vaccination campaigns were conducted based on the global positioning data. After vaccination, hospital surveillance of new cases was conducted to observe the effect of the methods used in mass immunization	At GI (G1+ G2 =188,206), *n* = 123,686 individuals were vaccinated with two doses of the vaccine. The fulldose was administered in 66%. Women were more involved, and 80% of the sample consisted of the vaccinated population (aOR: 1.80; 95% CI = 1.75-1.84) and younger people, under 15 years of age (aOR: 2, 19; 95% CI =2.13-3.26)	The instrument provides health managers with an understanding of the impact of the participation of different vaccine providers in immunization campaigns
Wilson et al., 2012, Canadá	Children aged 2, 4, 6, 12 and 18 months. N = No informations	Diphtheria, pertussis, tetanus, poliomyelitis, [Hib] (DaPT-IPV-Hib), measles, mumps, MMR and meningococcal C	VISION Data Systems (vaccine and immunization surveillance in Ontario)	They studied the safety of vaccines given to children in Ontario at 2, 4, and 6 months of age and then at 12 and 18 months of age	Reduction in events (mortality or morbidity rate) after mass vaccination. The event rate in the 3 days before vaccination was about half that of the event rate in the control period for the 4- and 6-month vaccinations	The strategy may help measure the impact of vaccination programs, but further studies are needed

*GI* intervention group, *GC* control group, *G1* group 1, *G2* group 2, *G3* group 3, *[Hib](DaPT-IPV-Hib)* Haemophilus influenzae type b, *MMR* Rubella

**Table 2:** Data extraction from randomized clinical trials

Study	Population	Intervention			Outcomes	
Study, year, country	Sample	Pathology	Instruments	Methodology	Prophylaxis	Technological progress in the health system
[32] 2017, Nigeria	Babies aged 0 to 3 months, in paired setting with their mothers. *N* = 595 infants. Intervention group (IG)= 295; control group (CG) = 300	Not stated	Questionnaires were used that addressed children’s immunizations and documented reminder activities for each participant. A weekly checklist was used to catch up and reschedule missed immunizations	The child’s primary caregiver received a reminder via cell phone call 2 days before the immunization and the day before. Children who were no-shows were automatically rescheduled for the next day	Increase in rate of compliance with immunization schedule by 79.2% and 46.6% in the control group (*p* < 0.001)	The use of electronic communication technology in public health interventions can improve immunization program adherence, promote health, and prevent disease
[19] 2017, Michigan	Adolescents aged 10–18 whose parents were recruited via promotional emails Season 1 sample GI = 888; GC = 101 Season 2 sample GI = 1.088; GC = 167	Influenza	Michigan Influenza Care Improvement Registry (MCR) is an app for tracking immunizations in children and adolescents < 20 years. The MCR was used to compare post-intervention data. Parents’ business email was used as a reminder to get vaccinated	Emails were sent in two flu seasons. During each season, 2 to 3 reminders were sent to patients, with an interval of 30 to 60 days between notifications. The child’s vaccination status was queried in the MCR before the reminder letters were sent out	Reminder in consecutive seasons was associated with a lower likelihood of influenza vaccination than those who received a reminder in only one season (aOR = 0.68, 95% CI = 0.55, 0.84). Adolescents eligible for influenza reminders in both seasons (*n* = 983 subjects; 548 in the notification group) were a subgroup that rarely received influenza vaccinations. Few (22%) were vaccinated in season 1 and 30% in season 2; only 14% of this subgroup received influenza vaccination in both seasons (*p* < 0.0001)	The use of technological tools to monitor and manage influenza vaccination could increase adherence. The ability to tailor reminders to patient preferences could increase responsiveness and have a greater impact on vaccination rates
[20] 2018, Denver	Adults aged 19–64 years who were not vaccinated with the vaccines studied. They were divided into group 1 = no risk and group 2 = high risk	Influenza, Tdap, and PPSV23	CIIS was the application used for vaccine monitoring. Reminders were sent by phone call or voice message	The IG, which consisted of patients classified as low risk, was contacted by reminders for approximately 3–4 months. Those who were not fully immunized against the vaccines analyzed were included	Although the results show that the CG had a higher probability of missing vaccination days compared to the low-risk population (2111/3301 68.1% intervention vs. 5510/8042 68.5% control, *p* = 0.65), (2928/4926 59.4% of intervention vs. 3022/4926 61.4% of control, *p* 0.05), no statistical differences were found between the two groups	No difference was observed after the intervention
[21] 2020, Louisville	Adults between 18 and 65 years. *n*= 50.286	Influenza	Participants were required to have the Humana Network Health Plan and the Human Wellbeing app installed. Reminders were sent via the app	Users registered vaccinations and received messages about vaccination reminders. Each time there was a user interaction in the immunization record, a scoring system was activated in the app. The messages sent were divided into 2 groups. In G1 = reward messages; G2 = vaccination reminders only; and G3 = no messages received	Of those who were to receive a message (G1 + G2, total *N* = 33.524), 7.764 (23.2%) received an influenza vaccination, compared with 3.696 (22.0%) individuals who were vaccinated in the control group. no message (G3). This difference was statistically significant (*p* < 0.01). There were no significant differences when comparing reward and no-reward messages	A mobile vaccination reminder platform may increase influenza vaccination rates by more than 1%. However, the effectiveness of the reminder decreased with each message sent, suggesting that participants who respond to reminders may be activated by receiving a single message
[22] 2015, New York	Adolescents aged 11–16 years who have not received HPV vaccination and whose parents have used public health services. *n* = 3.812	HPV	Reminders via text messages by managed care organization (primary care). Registration and immunization data were recorded in MCO software	39 primary care clinics were recruited, each enrolling more than 175 adolescents. After screening adolescents’ immunization data, the MCO programmer sent up to 04 text messages to remind them to bring their children for immunization. The GC consisted of messages that included general health guidelines but did not indicate the need for immunizations	In a post hoc analysis for all participants (i.e., all ages and both sexes) with valid phone numbers who did not opt out of receiving the introductory message, there was a 30% increase in HPV vaccination dose 1 (13% of the control group and 16% of the intervention group received a dose), controlling for age and sex (Table 3) (*p* = 0.04). There were no significant differences for doses 2 and 3 (*p* = 0.27 and *p* = 0.43, respectively)	The reminder system increased the vaccination rate of the intervention and control groups by more than 10%
[23] 2020, New York and Colorado	Adolescents aged 11–19 years New York *n* = 30.616 Colorado *n* = 31.502.	HPV	CIIS, NYSIIS. CIIS, NYSIIS. Message reminder system	A 4-arm, pragmatic, randomized controlled trial conducted in 2 states. Patients aged 11 to 17.9 years who had not completed their HPV vaccination series were randomly assigned to receive 0, 1, 2, or 3 IIS C-R/R autodial messages per vaccine dose	In Colorado, the 1-call R/R arm had higher rates of HPV vaccination initiation than the control arm in unadjusted analyzes. In adjusted analyzes, the 1-call R/R arm (adjusted RR = 1.07; 95% CI 1.04–1.10) and the 3-call R/R arm (adjusted RR = 1.04; 95% CI 1.01–1.06) had higher initiation rates than the control arm	C-R/R based on IIS autodial reports for HPV. Vaccination was not effective in increasing HPV vaccination rates in New York. C-R/R is low-cost software, but further studies are needed to find better strategies to increase vaccination rates

**Table 3:** Data extraction from technological products

Study	Population	Intervention				Outcomes	
Study, year, country	Target group	Programming platform	Methodology	Features and benefits	Limitations	Publication/evaluation	Incorporation/impact
[31] 2021, Malaysia	System and hospital administrator	Firebase web application interface and Firestore cloud—database. Metamask. Hyperledger Besu	Vaccination system with Blockchain. Method for storing vaccination record. Blockchain network. Functionality analysis with experts.	Immunization data distributed on sync nodes. Hospital has resources to present vaccination certificate and patient record	Full administrative control, including hospital rejection for linkage	Blockchain vaccination system—Hyperledger Besu. Connection between Hyperledger Besu and the cloud database	Not used. Control of basic registration and administrative vaccination data
[12] 2021, India	Guardian of children and authorities Child Vaccination	Smartphone/Android/Java/Firebase application Firestore and Firebase authentication	Analysis in experimental test. Installed on 10 smartphones. Simulation of the scenario	Calendar control; booking appointments in hospitals; sticky notes; messages; vaccination history and involvement of other children	Full administrative control, including hospital rejection for linkage	Users have agreed to the app and want the government to integrate it into the health care system	Do not. Manage immunization programs
[25] 2020, Iran	Public authorities/health authorities	Data from cellular contact tracing networks. SIR Model for the spread of disease	Vaccine allocation—per-population modeling and plots from contact tracking data derived from cellular networks and Bluetooth signals. Test on 236 school children	Contamination vaccine allocation model for outbreak control	Control of infected and uninfected	Targeted vaccination reduces the number of infected	Do not. Create a model of targeted vaccination in a cellular network
[17] 2015, United States	Public authority/health authorities	TelosB Crossbow/Wireless Sensor	Centrality algorithms for targeted vaccinations. Sample with students	Student contact tracing system. Graph of disease spread. Connectivity centrality metric algorithms based on targeted inoculation (targeted inoculation).	Use the model SIR, to simulate the infection process. Controlled scenario	Determined disease spread, created spread diagram for modeling	Do not. Obtained connectivity centrality metrics and disease spread and centralization algorithms
[13] 2021, South Korea	Authority/health authorities	Deep Learning; You Only Look Once Version 5 (YOLOv5s). Android and IOS	Immunization record management system and authentication routine—facial recognition. Mobile Vaccination Application (MVA)	Validation of the vaccination record using the Vaccination Information Management System (VIMS)	Cloud-based distributed database. Record of vaccination. Place of residence, city, date of vaccination, unique code, etc. Ability to verify mask usage and validate vaccination status and authentication	Average system accuracy of 99.5% for mask use. Need 100% vaccinated and unvaccinated people in real time	Not required. Ability to use this system on a large scale for industrial and commercial purposes
[18] 2013, United States	Children Rural area/government	Mobile application; Android SDK. We used SQLite; QR code processing	Cell phone application system for children vaccination control	System that stores user identification; type of vaccine; gender; QR associated with child’s ID.	Not validated in rural settings	They investigated the effectiveness of the system in terms of QR code sticker durability, robustness, and operating costs	Not tested. Potential for scalability and ease of use by medical staff
[26] 2018, Iran	Hospitals Health Care Professionals	Web and flowcharts interface	Effective electronic immunization registry system (EIRS)	Capture immunization data based on a complete form.	Captures demographic information such as age, sex, education, and place of work. Capture vaccine data Name of vaccine, route of administration, location, serial number of vaccine, country of manufacturer, and possible side effects. Generation of tables and charts	Pilot study. Difficulties in obtaining complete vaccine data	Not being conducted. Development of a digital immunization record for medical personnel in Iran

*SIR* Simple mathematical model where the population is divided into three groups = susceptible, infected, and recovered,* EIRS* effective electronic immunization registry system

**Table 4:** Data extraction from gray literature

Study	Population	Intervention				Outcomes	
Study year, country	Target group	Programming platform	Methodology	Features and benefits	Limitations	Publication/evaluation	Incorporation/impact
[30] 2019, Africa	Nurses, doctors, parents and school administration staff	Electronic systems for storing immunizations	Review data storage profiles in BRICS. Evaluate the use of a common electronic platform for accessing the Manual—by three segments paramedics, health care team, and responsible parties	Reduces information errors. Bidirectional flow. Control of immunization rates	Not implemented, just a proposal with impact analysis	In BRICS, only two countries have mixed control over digital and physical data: Brazil and Russia	Not implemented. Perception maps of replacing the current paper vaccination card with electronic vaccination
[24] 2019, Italy	A total of 21 articles with memory system and IIS	Immunization Information Systems (IIS)	Systematic review—benefits of using IIS as a tool to combat vaccination hesitancy	Automatic reminder/recall system, system interoperability, decision support system, website interface, and ability to capture adverse events after vaccination; groupings	The types of systems analyzed exclude apps	Assessment of use of systems of record	Not conducted. It concludes that there are insufficient studies to evaluate the effectiveness of IIS in addressing vaccine hesitancy
[27] 2019, Brazil	Not aplicable	PROimuni App	They developed and evaluated a mobile application on child vaccination. Test 22 nurse judges, 9 judges from the field of information and communication technologies; 22 mothers evaluated aspects such as: objectives, appearance, structure, usability, efficiency and interactivity	The app consists of 20 screens with functions such as: Immunization card, Vaccine information, Immunization schedule, Geolocation, Frequently asked questions	Extension of use and robustness of synchronization with the state system	Tool potential for vaccine management with cell phone	Not suitable. Proposal with ease of use and usability

*IIS* Immunization Information Systems,* IOS* Operational system

### Analysis bias risk

**Fig. 5:**
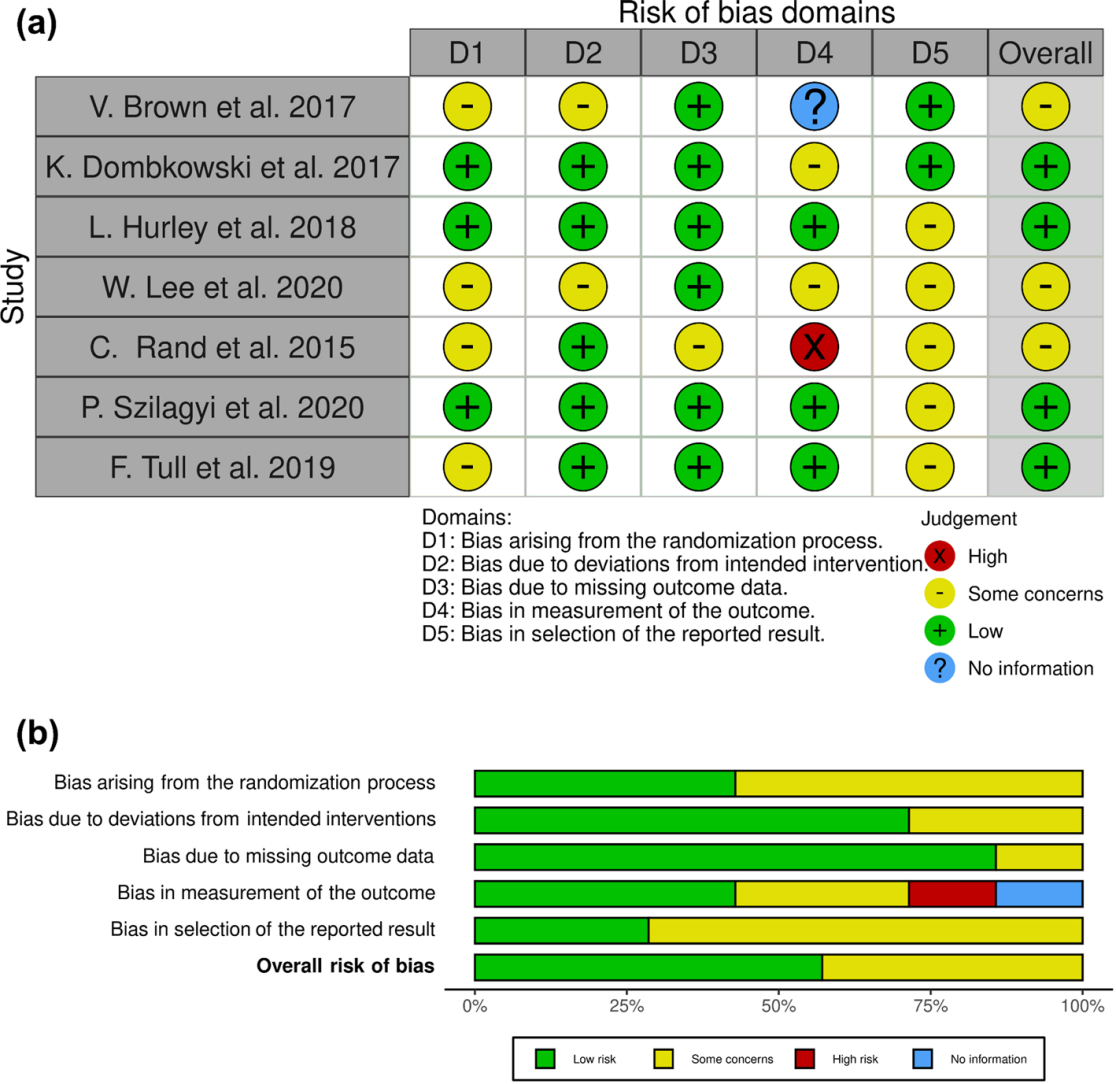
Cochrane risk-of-bias tool for randomized trials (RoB 2.0)

The seven studies were screened in all five domains, with [19, 20, 22, 29] being classified as high quality and low risk of bias by the algorithm used. The other studies [21, 22, 32] were included without sufficient information to confirm quality and risk. In Fig. 5b shows that the studies for areas 05, 04, and 01 each have uncertainties regarding quality.

**Fig. 6:**
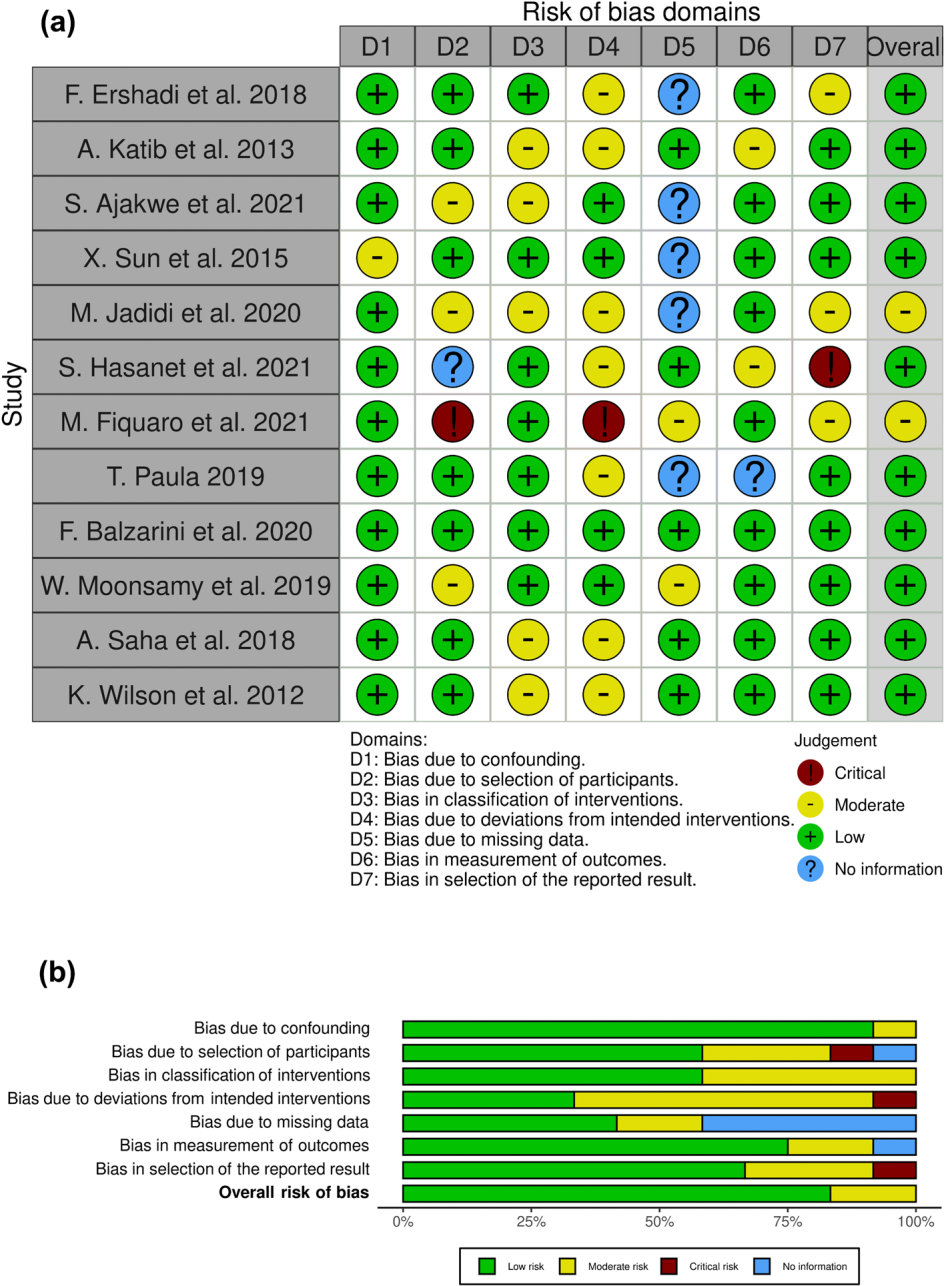
Risk-of-bias analysis performed with ROBINS-I (Risk of Bias in Non-randomized Studies-of Interventions), Cochrane

The other studies (*n* = 12) that did not have a clinical trial design, the ROBINS-I protocol tool was adopted. Figure 6a shows that ten of these studies were classified by the algorithm as being of low quality in terms of risk of bias. Only two studies [25, 31] have a moderate risk of bias. In terms of domains, Fig. 6b shows that domain 04 was the most problematic, followed by domain 05, which was unclear in two studies [30, 31], and another five did not have the information [13, 17, 25–27].

## Discussion

The immunization record is a health document (paper card) that records the immunizations received by a person. Traditionally, it has contained important data about the patient, such as the vaccine administered, the date of administration, the batch, the supplier, and other data required by the country’s health care system. Limited paper logistics can impact healthcare immunization strategies and affect safety, efficacy, and surveillance [34]. Digital immunization cards are immunization records in an electronic format that can be accessed by the patient and authorized health care personnel for the same purpose as the paper card: to ensure continuity of care or to provide proof of immunization. In addition, digital solutions can facilitate access to health data, streamline immunization campaigns, and provide greater security for data storage [34]. In 2020, the pandemic triggered by the SARS-CoV-2 coronavirus forced public health authorities around the world to propose social behavior restrictions, forms derived from the concept of contagion control understanding that some pathogenic biological entities can spread rapidly between individuals [7]. The studies selected for this review evaluated the use of digital vaccination records to examine their impact on disease prevention.

The history of immunization and disease eradication directly relates to attempts to create powerful vaccination programs. Several studies have attempted to improve, sustain, and support mass immunization strategies [12]. Although telemedicine did not emerge until the 1960s, it has gained worldwide prominence because of the disruption caused by COVID-19 and the resulting social isolation measures. In addition to telemonitoring, telemedicine has been used previously as an immunization strategy in the mapping, distribution, and monitoring of vaccine doses [35, 36]. Immunization information systems (IIS) are public health tools that store data on vaccination coverage at a given location. They offer many advantages in terms of identifying at-risk groups, managing, storing, and protecting data, and sharing information among health care providers when necessary [34].

In the selected articles, IIS were used to collect immunization data from individuals and contacts and to send reminders. The use of this technology can track and guide health managers on immunization strategies [19–23, 36]. Studies [19, 24, 30, 32] show that the use of technology and the collection of information and immunizations have a positive impact and that transmitting reminders and sending messages can provide additional awareness. Digital communication, educational messages, technical details, reminders, vaccination statements, phone numbers, and accessibility can be factors that contribute to the spread of vaccination.

Reminder systems were analyzed in four main areas: voice messages, text messages, calls, and emails. All selected studies, except the one conducted by Brown and Oluwatosin [32], used computerized platforms to register vaccinations from the public or private health system. In this work, patient data was collected using surveys and paper controls. The positive and statistically significant results in increasing adherence to the immunization program indicate that the computerization of health systems is necessary to optimize the outcomes of interventions [32]. In addition, the way in which reminder letters were sent to patients or caregivers was analyzed in several ways, including use of incentives [21]. The strategies with the lowest cost were voice messages and short phone calls. However, after comparing the results obtained, it was concluded that although the reminder system is effective, the way it reaches the patient needs to be adapted to individual preferences to be more effective. Therefore, more studies should be conducted that take into account the ease of access to information that globalization and technological advances have brought in recent years.

Tracking risk groups, the population to be immunized and the effectiveness of vaccine applications can also rely on technologies that use global positioning systems, as in the studies of [28, 33, 36]. The tools helped health professionals and provided feedback to managers on the number of hospital admissions and contamination of health service users following vaccination [28, 33]. Geolocation of individuals does not necessarily have to be done with expensive software. One of the largest studies of vaccination was conducted in the Congo to eradicate poliomyelitis used images taken with Google Earth. Despite the difficulties of not being able to update some of the satellite images, the study managed to suppress cases. It has become an example of low cost and accessibility for health professionals [36].

Other applications and software registered in coursework, theses and dissertations relate to registration control of patients [17, 18, 31] and health workers, management of immunization programs [12, 25] and registration and authentication of immunization records through facial biometrics [13]. Despite the potential use of these technologies for vaccine monitoring, little consideration has been given to the ethical protection of user data in applications, web systems and even wireless sensor networks, blockchain and QR codes. We noticed a lack of information or detail on how to ensure security and confidentiality, which we consider an important limitation of the technologies. We understand that review had limitations—the association of prophylaxis and the use of immunization control and health technologies greatly expands the scope—for example, we had 124 items in the first filter. In fact, we still have a lot to develop in terms of primary research in this scenario. For example, measures taken by government agencies to monitor important data related to immunization include the quality of data collected, the safety of samples and user feedback [13, 17, 27].

In Brazil, the Unified Health System (SUS) adopted the Conecte SUS application, with the goal of improving the exchange of health information during the transition of care and throughout care networks. Using cell phones or computers, citizens started to view their vaccination history, test results, prescription medications and other services offered by the national health system [37]. The use of technology to integrate health data not only provides patients with greater control over consultations, exams and disease prevention, but also allows for a faster response from the health professional when providing assistance [38]. The challenges faced in optimizing a digital health system revolve around the often difficult access to technology in certain regions of the country. Despite technical challenges, a proposal has been studied where part of the collections of vaccination record data are viewed offline, with the possibility of uploading later [39].

In summary, monitoring the vaccination process along the data chain, from manufacturing to the patient requires measures including many social actors, various technologies, effective communication, along with an established technological base. Another important actor in this context is also the end user, whose participation in maintaining his or her data updated is essential. Financial and social security monitoring are important examples of tools that have great potential influence and control. The integration of research and development in the innovation process is mediated for the technology in question by the integrative research model: the combination of the capacities of companies, the identification of opportunities, the development and accumulation of capabilities, and the science and broader technology in which they operate, which makes it possible to transfer the innovation process back to scientific phases to improve aspects inherent to market opportunities.

Within this context, there is a need to introduce here the translation that in the development of projects in fact promotes the development of new technologies, new practices of prophylaxis control and through public policy, support of the private sector, society and universities or research center are built—the creation of a quadruple helix [40] Indeed, it is essential that we respond to proposals that carry out complete and effective coverage of the immunization situation across a wide spectrum, as has been shown [26]. Even if a website [26] is an option that does not involve feedback and appropriation of the vaccinated individual. A system that fully manages the national immunization process can have a positive impact, from the production chain producing more vaccines in a given scenario, expanding health promotion, and on aspects of labor quality, minimizing attrition, and on making vaccines fully safe.

In this study, we did not look at the actions of anti-vaccinationists, but despite this challenge, a robust system moving in this direction, benefits could certainly be significant from increased awareness, education campaigns, and alignment across the board, as well as from protective laws. The limiting of breakdowns in health care systems should be a priority in the search for solutions in this area. We also emphasize that this study supports public policies that should recommend clinical management protocols, measures related to distribution, structures, batches, and traceability, and that light technological solutions applied to this scenario are a way forward identified by this review.

## Limitations

Several limitations were noted in the preparation of this systematic review. First, there was heterogeneity among the study proposals in finding a digital solution for vaccination. We had some studies that used different methods of information collection and had low validation in practice. The lack of a non-historical electronic record of vaccination or lack of updating of this makes it difficult to collect the sample in randomized clinical trials.

## Conclusions

Promoting action in public health, research, and disease control means promoting advances in basic and applied science. Failure, uncertainty, and ineffectiveness would be reduced, as would misinformation. We found that some of the articles analyzed here [30, 45] that presented reviews attempted to build this chain but were found to lack understanding of translation or technology maturity levels (TRLs), suggesting that the technology has reached a maturity level where systems/subsystems are developed in a necessary environment, i.e., in compliance with regulatory requirements. This is an important diagnosis made in this study. The creation of products, processes and/or services that have the potential to solve real problems and that affect the population proves the characteristic of the potential for technological and innovative production. We conclude that the mapping and control of vaccines are an area that needs to be researched and, in particular, that it is necessary to develop theories about the technology and its implementation chains, as well as about the effects of its prophylaxis.

## Methods

This study summarizes surveys conducted over the last decade, analyzing the effects of prophylaxis with control by the vaccination card. The study development methodology and inclusion and exclusion criteria are listed below.

### Protocol and registration

This study was conducted in accordance with PRISMA Preferred Reporting Items for Systematic Reviews and Meta-Analyses guidelines [41, 42]. The protocol for this systematic review was registered in the International Prospective Register of Systematic Reviews (PROSPERO) [43] under registration number: CRD42022298069.

We used the reference management software Mendeley® and divided the folders according to the databases consulted: CINAHL, IEEE Xplore, LILACS, PubMed/MEDLINE and Web of Science. Full reading of the title and abstract was done in pairs.

### Eligibility criteria

#### Inclusion criteria

Studies were considered that examined the use of digital vaccination books (P) to analyze the impact of a digital vaccination card on disease prophylaxis, conversion of the vaccination card to digital technology as a strategy to prevent disease and improve vaccination coverage (I), comparison of the impact on prophylaxis between vaccination control by a digital vaccination book (C), analysis of the impact on prophylaxis with control by a vaccination card (O). In this systematic review, inclusion criteria were based on the PICO (population, intervention, comparison, outcome) approach [44]. Articles published in the last 10 years (2012–2022), in Portuguese, English or Spanish, dealing with the prophylaxis of diseases susceptible to preventive vaccination were included.

#### Exclusion criteria

Several studies were excluded for the following reasons: (i) reviews, letters, personal opinions, book chapters and conference abstracts; (ii) in vitro and model animal studies; (iii) studies not fully published; (iv) duplicates; and (v) studies outside the time period (2011–2021).

### Information sources and search strategy

Individual search strategies have been developed for each of the following bibliographic databases: CINAHL, IEEE Xplore, LILACS, PubMed/MEDLINE and Web of Science. The strings customized for each database are listed in the Additional file 1. In addition to the 05 databases, we searched for similar studies in PROSPERO and the Cochrane Library, where we obtained no results. The search was performed between December 15, 2021, and February 11, 2022. Studies from the last 10 years in Portuguese, Spanish, and English were filtered out. Duplicate references were removed using Reference Manager software (Mendeley®).

### Study and selection

After performing the bibliographic search, we filtered articles that followed the study proposal and the acronym PICO. Thus, we performed a screening of the retrieved studies in two stages. In the first stage, six reviewers (Fleury Rosa, Silva, Santos, Silva, Perillo, Mendonça) independently evaluated the extracted articles from the databases, taking into account admissibility criteria established in this study (excluding studies that did not meet the inclusion criteria Additional file 3). In the second phase, the full texts of each article were obtained and evaluated by three pairs of authors (Fleury Rosa, Silva, Tatmatsu-Rocha, Carneiro, Oliveira, Rosa), selecting articles related to the predefined topics and/or subtopics. Finally, the data obtained was summarized in a “Results and Discussion” section written by the authors of this study in strict compliance with the PRISMA 2009 review protocol for systematic reviews [42].

### Risks of bias and quality in individual studies

We assessed the methodological quality of the 19 studies including analysis for the risk of bias using the Cochrane Collaboration Tool for Bias Risk Assessment. We assessed the quality of the studies by having four independent reviewers (Fleury Rosa, Silva, Santos, Silva) analyze the risk of bias of the included articles, and a fifth reviewer was consulted in case of divergent answers. Randomized clinical trials (*n* = 07) were analyzed using the RoB 2.0 platform (Additional file 2). The RoB 2.0 tool has 5 domains: (1) bias due to randomization process; (2) bias due to deviation from planned interventions; (3) bias due to missing outcome data; (4) bias in outcome measurement; and (5) bias in the selection of reported study [45]. Studies were categorized as “low” for low risk of bias, “high” for high risk of bias, “some concern” for uncertain risk, and “no information” for insufficient or missing information.

For the other included studies (*n* = 12), which are not considered clinical trials, we used the ROBINS-I (Risk Of Bias In Non-randomized Studies-of Interventions). It includes 07 domains: (1) confounding bias, (2) participant selection bias, (3) intervention classification bias, (4) deviation bias from planned interventions, (5) missing data bias, (6) outcome measurement bias, and (7) reported outcome selection bias. The scoring parameters were “low” for low risk of bias, “moderate” for moderate risk of bias, “high” if the risk of bias is severe, “critical” for critical cases of risk of bias, or “no information” for areas with no information. By rating the reviewers for each domain, it was possible to derive the overall quality of each study, and the respective results of this rating were presented using the Risk-of-bias VISualization (robvis) [46].

## Supplementary Information

**Additional file 1.** Database search and results.

**Additional file 2.** Risk of bias of selected individual studies.

**Additional file 3.** Excluded articles and reasons for exclusion.

**Additional file 4.** Characteristics of included studies.

**Additional file 5.** Glossary.

## Data Availability

The data sets used and/or analysed in the current study are available upon request from the corresponding author. The original papers presented in the study are included in the Article/Additional files. Further inquiries can be directed to the corresponding author.

## Abbreviations

WHO: World Health Organization
ICVP: International Certificate of Vaccination or Prophylaxis
ID: Identity
SUS: Unified Health System
NHDN: National Health Data Network
CIVP: Certificate of International Vaccination or Prophylaxis
ANVISA: National Health Administration
1D: One dimensional
2D: Two dimensional
GI: Intervention group
GC: Control group
G1: Group 1
G2: Group 2
G3: Group 3
[Hib](DaPT-IPV-Hib): Haemophilus influenzae type b
MMR: Rubella
MCR: Michigan Care Improvement Registry
Tdap: Triple bacterial vaccine used against tetanus, diphtheria, and pertussis
PPSV23: Pneumococcal vaccine
HPV: Human papillomavirus
COM: Monroe Plan Medical Care
CIIS: Colorado System—system of immunization sites in Colorado
NYSIIS: The New York State Immunization Information System
C-R/R: Centralized Reminder and Recall
IIS: State Immunization System
R/R: Reminder and Recall
SIR: Simple mathematical model where the population is divided into three groups = susceptible, infected, and recovered
EIRS: Effective electronic immunization registry system
IIS: Immunization Information Systems
IOS: Operational system
