# Supplementary material for: Effects of vaccine registration on disease prophylaxis: a systematic review
Source: Biomed Eng Online. 2022 Dec 3;21:84. doi: 10.1186/s12938-022-01053-z (PMC9719654; doi:10.1186/s12938-022-01053-z)
Supplement: Supplementary file 1 — Additional file 1. Database search and results. [file 12938_2022_1053_MOESM1_ESM.pdf]

Additional File 1 - Database search and results.

**Database**	Search	Quantity of publications
**WEB OF SCIENCE December 15, 2021, Update: Febray 11, 2022**	(((Immunization* AND Active) OR "Active Immunization*" OR "vaccination policy" OR "vaccination program*" OR vaccinotherapy OR "virus vaccination") AND (((App* AND Mobile) OR (App* AND "Portable Electronic") OR (App AND "Portable Software") OR (App* AND Portable) OR Software OR (App* AND “Portable Software”) OR (“Electronic App” AND Portable) OR "Mobile App*" OR "Mobile Application*" "Portable Electronic App*" OR "Portable Software App*" OR ("Software App*" AND Portable) OR "mobile app*" OR "portable software app*" OR (Application AND Mobile) OR (Application* AND “Portable Electronic”) OR (Application AND Portable) OR Software) OR (Application* AND “Portable Software”) OR (Apps AND “Portable Software”) OR (“Electronic Application*” AND Portable) OR "tablet application" OR iSpO2 OR "mobile health application" OR Moovcare OR Neomate) AND (((Card* AND "Patient Identification") OR (Card* AND Smart) OR "Health Smart Card" OR ("Identification Card*" AND Patient) OR "Patient Identification Card*" OR "Smart Card" OR ("Smart Card" AND Health) OR "Smart Cards" OR (Awareness* AND Vaccination) OR (Campaign* AND Vaccination) OR "Immunization Program" OR (Program* AND Immunization) OR (Promotion* AND Vaccination) OR "Vaccination Awareness*" OR "Vaccination Campaign*" OR "Vaccination Promotion*" OR "diagnostic services" OR "immunisation programmes" OR "immunization programmes" OR "needle-exchange program*" OR "preventive health services" OR "preventive service" OR (Coverage* AND Immunization) OR (Coverage* AND Vaccination) OR "Immunization Coverage*" OR "International Immunization Certificate" OR "International Vaccination Certificate" OR Prophylax* OR "Mass Vaccinations" OR (Vaccination AND Mass) OR "Mass Immunization*" OR (Immunization AND Mass) OR "refusal of vaccination" OR "vaccine refusal"))) AND ("Health Control of Travelers" OR "Obligatory Vaccination" OR "preventive therapy" OR (prevention AND control) OR "preventive measures" OR (prevention AND control) OR Immuni*ation OR "immunisation efficiency" OR "immunization efficiency" OR "immunisation prophylaxi*" OR "immunization prophylaxi*" OR "immunisation schedule" OR "immunization schedule" OR "immunostimulation therapy" OR "oral immuni*ation" OR reimmunization OR "standard immunization program" OR prophylaxis OR (prevention AND control) OR “prevention control” OR	1218

	"Communicable Disease" OR (Disease AND Communicable) OR "Infectious Disease*" OR (Disease* AND Infectious) OR Immunisation OR "standard immunization program")<br><br>#1 AND #11 AND #12 and 2012 or 2014 or 2013 or 2015 or 2017 or 2018 or 2016 or 2019 or 2020 or 2021 or 2022 (Anos da publicação) and Artigos or Artigos de revisão (Tipos de documento) and English or Spanish or Portuguese (Idiomas	
**MEDLINE/PUBMED December 15, 2021, Update: Febray 11, 2022**	("Vaccination"[MeSH Terms] AND ("Mobile Applications"[MeSH Terms] OR "Health Smart Cards"[MeSH Terms] OR "Immunization Programs"[MeSH Terms]) AND ("prevention and control"[MeSH Subheading] OR "Communicable Diseases"[MeSH Terms] OR "Immunization Schedule"[MeSH Terms])) AND ((y_10[Filter]) AND (clinicaltrial[Filter] OR meta-analysis[Filter] OR randomizedcontrolledtrial[Filter] OR review[Filter] OR systematicreview[Filter]) AND (english[Filter] OR portuguese[Filter]))<br><br>("Vaccination"[MeSH Terms] AND ("Mobile Applications"[MeSH Terms] OR "Health Smart Cards"[MeSH Terms] OR "Immunization Programs"[MeSH Terms]) AND ("prevention and control"[MeSH Subheading] OR "Communicable Diseases"[MeSH Terms] OR "Immunization Schedule"[MeSH Terms])) AND ((y_10[Filter]) AND (clinicaltrial[Filter] OR meta-analysis[Filter] OR randomizedcontrolledtrial[Filter] OR review[Filter] OR systematicreview[Filter]) AND (english[Filter] OR portuguese[Filter] OR spanish[Filter]))<br>Filters applied: Clinical Trial, Meta-Analysis, Randomized Controlled Trial, Review, Systematic Review, in the last 10 years, English, Portuguese, Spanish.	407
**LILACS/BSV September December 15, 2021, Update: Febray 11, 2022**	((((Immunization* AND Active) OR "Active Immunization*" OR "vaccination policy" OR "vaccination program*" OR vaccinotherapy OR "virus vaccination")) AND ((((App* AND Mobile) OR (App* AND "Portable Electronic") OR (App AND "Portable Software") OR (App* AND Portable) OR Software OR (App* AND "Portable Software") OR ("Electronic App" AND Portable) OR "Mobile App*" OR "Mobile Application*" "Portable Electronic App*" OR "Portable Software App*" OR ("Software App*" AND Portable) OR "mobile app*" OR "portable software app*" OR (Application AND Mobile) OR (Application* AND "Portable Electronic") OR (Application AND Portable) OR Software) OR (Application* AND "Portable Software") OR (Apps AND "Portable Software") OR ("Electronic Application*" AND Portable) OR "tablet application" OR iSpO2 OR "mobile health application" OR Moovcare OR Neomate)) AND ((((Card* AND "Patient	04

	Identification") OR (Card* AND Smart) OR "Health Smart Card" OR ("Identification Card*" AND Patient) OR "Patient Identification Card*" OR "Smart Card" OR ("Smart Card" AND Health) OR "Smart Cards" OR (Awareness* AND Vaccination) OR (Campaign* AND Vaccination) OR "Immunization Program" OR (Program* AND Immunization) OR (Promotion* AND Vaccination) OR "Vaccination Awareness*" OR "Vaccination Campaign*" OR "Vaccination Promotion*" OR "diagnostic services" OR "immunisation programmes" OR "immunization programmes" OR "needle-exchange program*" OR "preventive health services" OR "preventive service" OR (Coverage* AND Immunization) OR (Coverage* AND Vaccination) OR "Immunization Coverage*" OR "International Immunization Certificate" OR "International Vaccination Certificate" OR Prophylax* OR "Mass Vaccinations" OR (Vaccination AND Mass) OR "Mass Immunization*" OR (Immunization AND Mass) OR "refusal of vaccination" OR "vaccine refusal")))) AND (("Health Control of Travelers" OR "Obligatory Vaccination" OR "preventive therapy" OR (prevention AND control) OR "preventive measures" OR (prevention AND control) OR Immuni*ation OR "immunisation efficiency" OR "immunization efficiency" OR "immunisation prophylaxi*" OR "immunization prophylaxi*" OR "immunisation schedule" OR "immunization schedule" OR "immunostimulation therapy" OR "oral immuni*ation" OR reimmunization OR "standard immunization program" OR prophylaxis OR (prevention AND control) OR "prevention control" OR "Communicable Disease" OR (Disease AND Communicable) OR "Infectious Disease*" OR (Disease* AND Infectious) OR Immunisation OR "standard immunization program"))	
**CINAHL Database/EBSCO December 15, 2021, Update: Febray 11, 2022**	(AB ((Immunization* AND Active) OR "Active Immunization*" OR "vaccination policy" OR "vaccination program*" OR vaccinotherapy OR "virus vaccination")) AND (S1 AND S2 AND S3)	86
**IEEE Xplore December 15, 2021, Update: Febray 11, 2022**	(((((("Document Title":Vaccinations) OR ("Document Title":vaccination program*) OR ("Document Title":vaccine?) AND ("Document Title":mobile health application) OR ("Document Title":Health Smart* Card*) OR ("Document Title": App*) AND ("Abstract":preventive health service*) AND ("Abstract":International Immunization Certificate) AND ("Abstract":International Vaccination Certificate or Prophylaxis) AND ("Abstract":Health Control of Travelers) OR ("Abstract":mmunisation schedule)))))) Filters Applied: Conferences, Journals, 2012 - 2021	113

**Total**		1828

Filters used: research from the last ten years (2011-2021) and articles.
