# Supplementary material for: Effects of vaccine registration on disease prophylaxis: a systematic review
Source: Biomed Eng Online. 2022 Dec 3;21:84. doi: 10.1186/s12938-022-01053-z (PMC9719654; doi:10.1186/s12938-022-01053-z)
Supplement: Supplementary file 2 — Additional file 2. Risk of bias of selected individual studies. [file 12938_2022_1053_MOESM2_ESM.pdf]

Additional file 2 - Risk of bias of selected individual studies.

**Criteria used for publication risk analysis**
1. Are experimental groups comparable and are control groups available?<br>2. Is the number of experimental units in each group clearly indicated? And is itthe same number (n) that was evaluated in the statistical analyses?<br>3. Were pre-established criteria for inclusion or exclusion of experimental unitsdescribed during the experiments or during the analyses?<br>4. Is there a description of the use of a method to blind researchers, especiallythose responsible for handling the data and analyzing the results?<br>5. Were the variables used to measure the results (conclusions) clearlydescribed?<br>6. Were details of the statistical analysis used in each analysis provided?<br>7. Has all relevant information about App features, technologies and systemsbeen clearly described?<br>8. Are the steps of the collection procedures and their intervals andmeasurements clearly described and detailed enough to allow replication?<br>9. Do the results include data from all data clearly described with an indicationof the value of the statistically significant difference (p-value)?<br>10. Is the study summary clear and does it include all relevant information? Likeobjectives, system using, impact, main methods used and relevant results?<br>11. Does the introduction provide information that contextualizes and justifies thedevelopment of the study?<br>12. Is the research question clearly described in the study objectives?<br>13. Is there a description of approval by the ethics committee and was the name ofthe committee informed?

14. Have the conditions of data collection, locations, participants and focus disease – type of vaccine been defined?
15. Have procedures been described for using the proposed solutions, technical details and processing?
16. Is the interpretation of results related to the objectives of the study? And were the limitations of the study described?
17. Is there a description of the results that indicates the possibility of transferring the technology for large-scale use, especially for prophylactic control?
18. Is there information about a study protocol that was developed prior to the start of experiments? If so, is there an indication of where it was published?
19. Did the study provide raw data on outcomes? Note: If this item is not present, the study should not be considered at high risk of bias, but this information is not available.
20. Is there a statement about the presence or absence of conflicts of interest in thestudy?

ROB 2.0 platform.
