# Supplementary material for: Effects of vaccine registration on disease prophylaxis: a systematic review
Source: Biomed Eng Online. 2022 Dec 3;21:84. doi: 10.1186/s12938-022-01053-z (PMC9719654; doi:10.1186/s12938-022-01053-z)
Supplement: Supplementary file 3 — Additional file 3. Excluded articles and reasons for exclusion. [file 12938_2022_1053_MOESM3_ESM.pdf]

Additional file 3 - Exclused items and rasons for exclusion

**Reason for Exclusion**	**Quantity**
1. Technological resources were not used for the immunization program	43
2. Technology used is not aimed at vaccine control devices	6
3. The study carried out is not in humans	3
4. It is about the development or testing of the effectiveness of a vaccine	3
5. It is at odds with the research topic:	3
6. Narrative Review	2
7. Excluded due to technical problems	10
**Total**	70
