# Supplementary material for: Effects of vaccine registration on disease prophylaxis: a systematic review
Source: Biomed Eng Online. 2022 Dec 3;21:84. doi: 10.1186/s12938-022-01053-z (PMC9719654; doi:10.1186/s12938-022-01053-z)
Supplement: Supplementary file 4 — Additional file 4. Characteristics of included studies. [file 12938_2022_1053_MOESM4_ESM.pdf]

Additional file 4 - Characteristics of included studies

Author, Year/	**Country**	**Sample**	**Population**	**Intervention**	**Control**	**Outcomes**
K. Wilson *et al.*, 2012	Canada	Data set based on direct abstraction from Ontario Health Insurance Program tables.	Children aged 2, 4, and 6 months and children aged 12 and 18 months.	Using the combination of linked data-subjects and vaccine types administered-from the Ontario Health Insurance Program, the safety of vaccines administered to children during this period was assessed.	The rate of events or hospitalizations of children before vaccination.	Primary outcome: we observed that in the analysis period of vaccinations, there was a decrease in emergency department events and hospitalizations 3 days after vaccination at 2, 4, and 6 months, which was a consequence of the healthy vaccination effect. At 12 and 18 months after vaccination, we observed that the number of emergency department visits increased approximately 1 week after dose administration. Secondary outcome: Collaboration with the Canadian surveillance system ACTive Immunization Monitoring Program (IMPACT) allowed validation of specific diagnoses identified from administrative health data linked to vaccinated and unvaccinated individuals. A population-based assessment of vaccine safety can provide an estimate of attributable risk and population impact. The correlation between the date of vaccination and the date of subsequent health care utilization suggests a lower risk of bias.
Peter Szilagyi *et al.*, 2020	United States of America	Adolescents with incomplete HPV vaccination series - in two regions: NY and Colorado.	Adolescents aged 11 to 17 years and children aged 9 years.	Patients were randomly assigned to receive 0, 1, 2, or 3 automated calls to remind them of vaccination. A	Do not send reminders to parents.	No significant effect of IIS-based CR /R autodialer calls was found on HPV vaccine series initiation or completion rates in New York and only small effects in Colorado for series initiation and completion with 1 or 3 CR /R vaccines. These largely negative results were inconsistent with

				telephone company sent automated calls to the main family telephone number to test the effects of automated C-R/R calls using only IIS data to increase HPV and vaccination rates.		hypotheses, given the success of this type of C-R/R for childhood vaccines, which was noted positively in the 2018 Cochrane report for vaccines.
W. Moonsamy *et al.*, 2019	Continent Africa - BRICS Countries	Design of a prototype for an electronic vaccination passport system.	Nurses (manage and document immunization records).<br>Physicians (manage and document immunization records) -Parents (keep immunization records secure) -School administrators (process immunization records when minors are admitted to school).	Data collection in BRICS countries - to verify exclusive use of a physical storage medium, such as a card to store records.	Not applicable.	There are advantages to using a digital system to store and manage immunization records. Research on BRICS countries also shows that 2 out of 5 BRICS countries successfully use a mix of digital and physical immunization records.<br><br>Another conclusion is how key stakeholders view the replacement of the current paper immunization card with electronic immunization?
A. Katib *et al.*, 2013	United States of America	Data sets from 2008 to 2011 from the National Immunization Survey.	Children aged 19 to 35 months.	A set of immunization records from 102,508 children was uploaded to the Jeev app, which tracks vaccination coverage. The vaccines studied were DTaP, poliovirus vaccine, MMR vaccine, Hib, Hep A, Hep B, varicella-zoster vaccine, PCV, and influenza vaccine.	Not applicable.	The authors conclude that Jeev is capable of collecting data from multiple clients and synchronizing them in real time on the server, while also highlighting its low cost. The system's performance and power consumption have only been tested with databases, and they intend to expand it to Haiti and India.

S. Ajakwe *et al.*, 2021	South Korea		Vaccinated and unvaccinated people, with or without masks.	Technological approaches and a compatible system for real-time monitoring and application of COVID -19 vaccination and prevention guidelines required data and QRcode authentication technology.	Not applicable.	The developed system achieves 95% real-time recognition, classification, authentication, validation, and application accuracy with lower computational requirements. This tool can be installed in banks, shopping malls, schools, or any other facility that requires crowd control. It achieved 99.5% accuracy in mask detection and 100% accuracy in real-time verification of vaccination status from COVID -19.
X. Sun *et al.*, 2015	United States of America	Students at a high school	800 students	A student contact tracing system was developed and implemented in a high school based on wireless sensors worn by students.	Not applicable.	The wireless sensor system allowed student contacts to be found within the disease spread distance and a graph of disease spread to model the spread of infectious diseases.
M. Jadidi., *et al* 2020	Iran	Data set on cumulative networks in elementary schools, i.e., a network for identifying contacts in elementary schools.	Daily face-to-face contacts between 236 individuals.	Prevention of the spread of COVID -19 through simulations and models. First, vaccines were distributed among the different communities of a society and, in a second step, the individuals to be vaccinated in each community were determined.		The simulations show that the combined metric of infection and susceptibility to infection can control the spread of the disease faster than any other metric alone, achieving a 30% decrease in the rate of infection compared to random vaccination.

S. Hasan *et al*., 2021	Índia	Tests on about 10 smartphones	Indian child population	'e-Vaccine' app to facilitate the immunization process and help parents and caregivers keep their children's immunization schedules up to date.	Not applicable.	Verified that the application was functioning correctly according to expectations.<br>The entire development of the application, including planning and testing, took approximately 45 days. Users who participated in the testing process found the app useful and commented on a simple interface that helps them better understand the immunization routine.
M. Fiquaro *et al*., 2021	Malaysia	The system test for evaluating the -Blockchain Vaccination System- 1 Black Box: visual system test; 2) White Box - system procedure.	Developers and users/administrators	Perform the analysis of a blockchain/web system. As for the user - login, page navigation, form submission and masking. As for the developer and administrator of the likely, it collected data of interest.	Not applicable.	The system application can be run as expected. The system has basic registration and immunization data for the blockchain proposal - the results indicate an open use opportunity to introduce more research on blockchain as a distributed system technology.
V. Brown *et al*., 2017	Nigeria	305 children were enrolled in the intervention group and 309 children in the control group.	4 to 1 randomly selected children did not receive the reminder intervention, and the remaining 3 received reminders for children 0 to 3 months of age.	To introduce a cell phone reminder program to improve adherence to routine immunizations among infants and vaccination coverage among young children. Mothers in the intervention group received a cell phone reminder two days before the child's next immunization	The control group received usual care.	They show that the use of client reminderand recall systems can provide community health nurses and other public health professionals with a real-world experience with community-based practice that can improve the health of the populations they serve.<br><br>The use of electronic communication technology in public health interventions can improve adherence to treatment, health promotion, and disease prevention guidelines.

				appointment and a second call the day before the appointment.		
K. Dombkowski *et al.*, 2017	United States of America	Patients aged 10 to 18 years from 4 practices in Michigan. Step 1 notified group = 888 records. Nonnotified group = 609 records. Step 2. notified group = 1088 records. Total of 1088 records. Group not notified = 960 records.	The records contained a unique combination of the child's name and the parent's email address. We only included emails from parents in the 11-17 year old subgroup. During each flu season, 2 to 3 e-mail reminders were sent to the clinic, with 30 to 60 days between each notification.	To examine the feasibility of emailing seasonal influenza vaccination reminders to parents of adolescents and (2) to evaluate the rate of influenza vaccination among adolescents whose parents were or were not randomly assigned to receive an email reminder. The primary outcome of the study was the detection of 1 new influenza vaccination dose recorded in the MCIR, with the date of administration of the dose within 60 days of the date of the email notification.	Not applicable	Influenza vaccination status of study participants was assessed using the MCIR for vaccines administered throughout the study period. While it is possible that the MCIR may not necessarily reflect all doses administered, Michigan state law requires that all doses for persons under 21 years of age be reported to the MCIR, and it has been shown to have a high degree of completeness.<br>It was not possible to analyze the results as a randomized clinical trial, and the characteristics of households affected by reclassification into the reported group were not available for our analysis.
L. Hurley et al., 2018	United States of America	15,769 - aged between 19and 64 years. Experimental (n = 7,612) and control (n = 7,541) For over-65s, experimental (n =	Patients were registered in the Colorado Immunization Information System (CIIS). Patients with usual care were selected by simple stratified random sampling.	Randomized, stratified, controlled trial of patients. It consisted of planned exit interviews to enrich understanding and interpretation of the controlled and randomized trial. All	The control arm received usual care, which did not include reminders from study staff about immunizations.	The primary outcome of the study was documentation of receipt of one of the two or three required vaccines in the CIIS within six months of R/R initiation. This outcome may include receipt of a vaccine or documentation of a previously received vaccine in CIIS because of efforts to complete the survey (average 6 months for full enrolment).

		307) and control (n = 309).		participants received Centers for Disease Control and Prevention posters in English and Spanish.		There were no significant differences between the two age groups in baseline characteristics or update rates for vaccines of interest.
W. Lee *et al.*, 2019	United States of America	50,286 individuals between the ages of 18 and 65. They were continuously enrolled in a health plan. They were active users of the Humana Wellness mobile app.	Adults aged 18 to 65 eligible for flu vaccination.	The study is the first randomized clinical trial to examine the effect of a large-scale information campaign combined with individual incentives on influenza vaccination rates.	No message	The primary outcome was influenza vaccination documented by physician and pharmaceutical inquiries to the health insurer, or when participants provided evidence of vaccination. Statistically significant benefit of using a mobile platform to get people vaccinated against influenza at the population level. Given the trend among healthcare providers and payers to use mobile technologies to engage and interact with patients, we believe these findings could have important implications for how these technologies can be used to promote and influence healthy behaviors at the population level.

Desenvolvimento de aplicativo móvel sobre vacinação infantil para pais TT - Development of a mobile app on childhood vaccination for parents<br><br>**Paula, Thais Rodrigues. (2019), Dissertation, Brazil**<br><br>(Poe nome da autora?)	Brazil	451 respondents (18.1%), out of a total of 2489 users at the time of application of the usage questionnaire.	From volunteers with different cell phone models.	Creation of a mobile application aimed at educating health professionals and patients.<br>For each vaccine included in the application, the corresponding data must be collected.	Not applicable.	Applying current medical informatics and telemedicine knowledge and leveraging the wide availability of mobile devices are strategic tools to improve vaccination coverage.
C. Rand *et al.*, 2015	United States of America	278 from the control group and 205 from the intervention group.<br>Messages were sent to the parents of 3,812 teens.	39 family practices (29 pediatricians and 10 family physicians) associated with the MCO - (managed care organization).	Through the MCO, they received health messages from their children's insurance company: 1st message, "MPHealth: [...] [name of insurance plan] THX! Message and data rate. 2nd message, (MPHealth: 'Your [age] should receive the HPV vaccination. Please call [phone number and name of practice] to make an appointment.	Parents in the control group received the same first message, followed by a control message on a different general adolescent health topic, each time a reminder was sent to the intervention group.	Rates of single-dose HPV vaccination were 16% at the end of the study, compared with 13% in the control group, a modest improvement. It is noteworthy that these rates are lower than national averages because we selected only adolescents who had never been vaccinated at the start of the study.<br><br>The study suggests that interventions in physician offices are also needed to dramatically improve HPV vaccination rates.

A. Saha et al., 2018	Bangladesh	268.896 participants	Of the 268.896 people living in the 90 clusters, 95.115 were in the vaccine group, 93.091 were in the vaccine and behavior modification group, and 80.690 were in the no intervention group. The OCVs were divided between the vaccine and vaccine-plus-behavior modification arms and included 60 groups with 188.206 people.	The vaccination program was designed and implemented using a geographic information system (GIS). The study consisted of three parts: (i) vaccine, (ii) vaccine plus behavior change component (BCC), and (iii) no intervention, and the design included 30 groups in each arm of the study.	Not applicable.	Of the 188.206 subjects in the intervention arms, 123.686 received two full doses of OCV (vaccinated) and 64.520 received one or no dose of the vaccine (unvaccinated), representing 66% coverage of full dose recipients. A total of 46.153 households were included in the study, with an average of 4 members in each household.
V. Gianfredi *et al.*, 2019	Italy	22 manuscripts were included in this study	Narrative review, as a comprehensive qualitative synthesis of previously published information.<br>The included articles met the following inclusion criteria: (1) full text available; (2) articles that used IIS as a primary data source; and (3) articles that focused on useful IIS features to combat HV (as described above).	U IIS as a tool to combat HV.<br>The primary focus was on data that were critical to answering the questions.	Not applicable.	Some of the potential uses of IIS include: Data on the characteristics of vaccination refusers, such as geographic distribution; knowledge of personal vaccination status, which seems to be much more important in the current context of migration/globalization and social mobilization; data on possible adverse effects after vaccination, which increase confidence in the vaccine.<br>The use of the IIS is a promising tool that is useful for vaccine providers and recipients as well as for health policy makers and epidemiologists.
F. Ershadi *et al.*, 2018	Iran	50	Health experts	Develop the first EIRS for use at Namazi Hospital, Shiraz, Iran.<br><br>They used sequences of events from the	Not applicable.	Effective and reliable recording could help reduce the risk of occupational disease transmission.<br><br>There have been difficulties in capturing a complete immunization history, developing registration software, and establishing guidelines

				arrival of the medical staff to the decision and follow-up of the vaccination.		for transferring information from paper forms to the electronic record.<br><br>Despite these challenges, we designed and developed a digital immunization record for medical personnel in Iran for the first time.
