# Supplementary material for: Effects of vaccine registration on disease prophylaxis: a systematic review
Source: Biomed Eng Online. 2022 Dec 3;21:84. doi: 10.1186/s12938-022-01053-z (PMC9719654; doi:10.1186/s12938-022-01053-z)
Supplement: Supplementary file 5 — Additional file 5. Glossary. [file 12938_2022_1053_MOESM5_ESM.pdf]

Additional file 5 - Glossary

BLOCKCHAIN VACCINATION SYSTEM: provides real-time visibility of vaccine distribution and a chain of record from manufacture to use.

BRICS: is a term used to describe the group of emerging economies consisting of Brazil, Russia, India, China and South Africa.

COMPUTED TOMOGRAPHY: a form of tomography in which a computer controls the movement of the X-ray source and detector, processes the data and produces an image.

CONNECT SUS: is a federal government programme coordinated by DATASUS in partnership with the end sectors of the Ministry of Health, whose mission is to implement the Digital Health Strategy for Brazil.

COVID-19 : is an infectious disease caused by the SARS-CoV-2 virus.

CINAHL: Cumulative Index to Nursing and Allied Health

CIIS: Colorado System - system of immunization sites in Colorado.

COM: Monroe Plan Medical Care.

C-R/R: Centralized Reminder and Recall. (IIS): State Immunization System.

EUROPEAN COMMISSION: is the executive component of the European Union. This means that it is responsible for initiating legislation, implementing EU law and managing EU policies.

GPS: global positioning data

HPV (Human Papillomavirus): is the most common sexually transmitted infection in the world.

ID: the identity that each user.

INTERNATIONAL PROSPECTIVE REGISTER OF SYSTEMATIC REVIEWS (PROSPERO): is an open-access online database of systematic review protocols on a variety of topics.

IEEE: Institute of Electrical and Electronic Engineers.

IT: integration of information technology.

MENDELEY: software for managing references.

MCO: managed care organization.

MICHIGAN CARE IMPROVEMENT REGISTRY: is an immunisation database that records Michigan residents' immunisations over the course of their lives.

National Health Data Network (RNDS): is the Brazilian national platform for interoperability (data exchange) in health. It is not only a structuring project of Connect SUS, but also a programme of the Brazilian government aimed at the digital transformation of healthcare in Brazil.

NYSIIS: The New York State Immunization Information System.

PICO: is an acronym for "Patient", "Intervention", "Comparison" and "Outcomes".

PRISMA: is an evidence-based set of elements for reporting studies in systematic reviews and meta-analyses.

SARS-CoV-2: A virus that causes a respiratory disease called coronavirus 19 (COVID19). SARSCoV2 belongs to a large family of viruses known as coronaviruses. These viruses can infect humans and some animals.

SISTEMA ÚNICO DE SAÚDE (SUS): It consists of the totality of all health activities and services provided in Brazil by public institutions and organisations.

DAS INTERNATIONAL CERTIFICATE OF VACCINATION OR PROPHYLAXIS (ICVP): also known as Carte Jaune or Yellow Card, is an official vaccination card issued by the World Health Organisation (WHO). As a travel document, it is an internationally recognised medical passport that may be required for entry into certain countries with increased health risks for travellers.

PPSV23: Pneumococcal vaccine.

R/R: Reminder and Recall.

ROBINS-I: (Risk Of Bias In Non-randomized Studies—of Interventions)

Tdap: Triple bacterial vaccine used against tetanus, diphtheria, and pertussis.

VOSviewer: is a software tool for creating and visualising bibliometric networks.

WORLD HEALTH ORGANISATION (WHO): part of the United Nations that addresses the world's major health problems. The World Health Organisation sets standards for disease control, health care and medicine, conducts education and research programmes and publishes scientific articles and reports.
